# Improved dosimetric accuracy with semi‐automatic contour propagation of organs‐at‐risk in glioblastoma patients undergoing chemoradiation

**DOI:** 10.1002/acm2.12758

**Published:** 2019-10-31

**Authors:** Sangjune Lee, James Stewart, Young Lee, Sten Myrehaug, Arjun Sahgal, Mark Ruschin, Chia‐Lin Tseng

**Affiliations:** ^1^ Department of Radiation Oncology Sunnybrook Odette Cancer Centre University of Toronto Toronto ON Canada

**Keywords:** automation, contouring, glioblastoma, MR-guided radiotherapy, MR-linac, organs at risk, propagation

## Abstract

**Background:**

We study the changes in organs‐at‐risk (OARs) morphology as contoured on serial MRIs during chemoradiation therapy (CRT) of glioblastoma (GBM). The dosimetric implication of assuming non‐deformable OAR changes and the accuracy and feasibility of semi‐automatic OAR contour propagation are investigated.

**Methods:**

Fourteen GBM patients who were treated with adjuvant CRT for GBM prospectively underwent MRIs on fractions 0 (i.e., planning), 10, 20, and 1 month post last fraction of CRT. Three sets of OAR contours — (a) manual, (b) rigidly registered (static), and (c) semi‐automatically propagated — were compared using Dice similarity coefficient (DSC) and Hausdorff distance (HD). Dosimetric impact was determined by comparing the minimum dose to the 0.03 cc receiving the highest dose (D0.03 cc) on a clinically approved reference, non‐adapted radiation therapy plan.

**Results:**

The DSC between the manual contours and the static contours decreased significantly over time (fraction 10: [mean ± 1 SD] 0.78 ± 0.17, post 1 month: 0.76 ± 0.17, *P* = 0.02) while the HD (*P* = 0.74) and the difference in D0.03cc did not change significantly (*P* = 0.51). Using the manual contours as reference, compared to static contours, propagated contours have a significantly higher DSC (propagated: [mean ± 1 SD] 0.81 ± 0.15, static: 0.77 ± 0.17, *P* < 0.001), lower HD (propagated: 3.77 ± 1.8 mm, static: 3.96 ± 1.6 mm, *P* = 0.002), and a significantly lower absolute difference in D0.03cc (propagated: 101 ± 159 cGy, static: 136 ± 243 cGy, *P* = 0.019).

**Conclusions:**

Nonrigid changes in OARs over time lead to different maximum doses than planned. By using semi‐automatic OAR contour propagation, OARs are more accurately delineated on subsequent fractions, with corresponding improved accuracy of the reported dose to the OARs.

**Abbreviations**
CBCT: cone‐beam CT
CRT: chemoradiation therapy
CT: computed tomography
D0.03cc: minimum dose to the 0.03 cc receiving the highest dose
DSC: dice similarity coefficient
FFE: Fast Field Echo
GBM: glioblastoma
HD: Hausdorff distance
MRI: magnetic resonance imaging
MRL: MRI‐linear accelerator
OAR: organ‐at‐risk

## 1 INTRODUCTION

Glioblastoma (GBM) is the most frequent primary malignant brain tumor in adults with an overall survival of 5.1% at 5 years from time of diagnosis despite aggressive therapy. In appropriate patients, current standard of care is maximal safe resection, followed by adjuvant concurrent chemoradiation therapy (CRT) to a total of 4000–6000 cGy.^1^, ^2^ During the 4–6 weeks between surgery and the initiation of adjuvant CRT, approximately 50% of patients develop tumor growth or changes in the resection cavity contrast enhancement pattern.^3^ In a computed tomography (CT)‐based imaging study, the median resection volume reduction was approximately 35% at week 4 of treatment.^4^, ^5^

Definition of the tumor and surrounding organs‐at‐risk (OARs) can be better visualized with magnetic resonance imaging (MRI) compared to CT. During treatment, cone‐beam CT (CBCT) images can show bony anatomy for positional alignment, but visualization of any changes to the tumor or OARs is limited. The current clinical workflow on CBCT‐enabled devices assumes that the interfractional changes in OARs are minimal and the planned dose distribution is reflective of the entire delivered dose. In addition to the postoperative MRI used for radiation planning purposes, additional MRIs are done as clinically indicated to investigate worsening symptoms and for routine follow‐up after completion of chemoradiation therapy. Recent development of hybrid MRI‐linear accelerator (MRL) systems could improve the precision of radiation treatment through daily MR imaging and plan adaptation. Use of the MRL is a natural extension of the existing routine planning process for GBM radiation in which MRI is fused to the planning CT. However, several challenges in workflow strategies, including the timely generation of new target and OAR contours for plan adaptation each day, need to be addressed before the MRL can be used clinically.^6^ There can also be considerable inter‐observer variability in the contours for different OARs. For example, one study suggests that the inter‐observer Dice similarity coefficient (DSC) for the brainstem is 0.83 and the DSC for the optic nerves is 0.5.^7^

The purpose of this study is to investigate the changes that occur in GBM OARs on serial prospectively acquired MRIs during adjuvant CRT. We investigate the accuracy and dosimetric implication of assuming non‐deformable OAR changes, in addition to the feasibility of semi‐automatic contour propagation for the purpose of an adaptive workflow in MR‐guided radiotherapy.

## 2 MATERIALS AND METHODS

### 2.1 MRI schedule and parameters

Under an institutional research ethics board approved research protocol, between June 2016 and January 2017, patients who have undergone a surgical resection for GBM were recruited to undergo prospective serial multiparametric MRIs on fractions 0 (i.e., planning), 10, and 20 of CRT and 1 month post last radiotherapy fraction. All images were acquired on a 3T Philips Achieva scanner (Philips, Best, Netherlands) and we studied the T1‐weighted post‐Gadolinium MRI, which consisted of a full 3D acquisition using the Philips Fast Field Echo (FFE) gradient echo sequence (TR = 9.5 ms, TE = 2.3 ms) with a voxel size of 0.49 × 0.49 × 1.50 mm.

### 2.2 OAR contouring

OARs including the brainstem, globes, optic nerves, and chiasm were manually contoured on each MRI by a senior radiation oncology resident (SLL) and verified by a board‐certified radiation oncologist (CLT). These contours served as a ground truth for contour evaluation.

**Figure 1:**
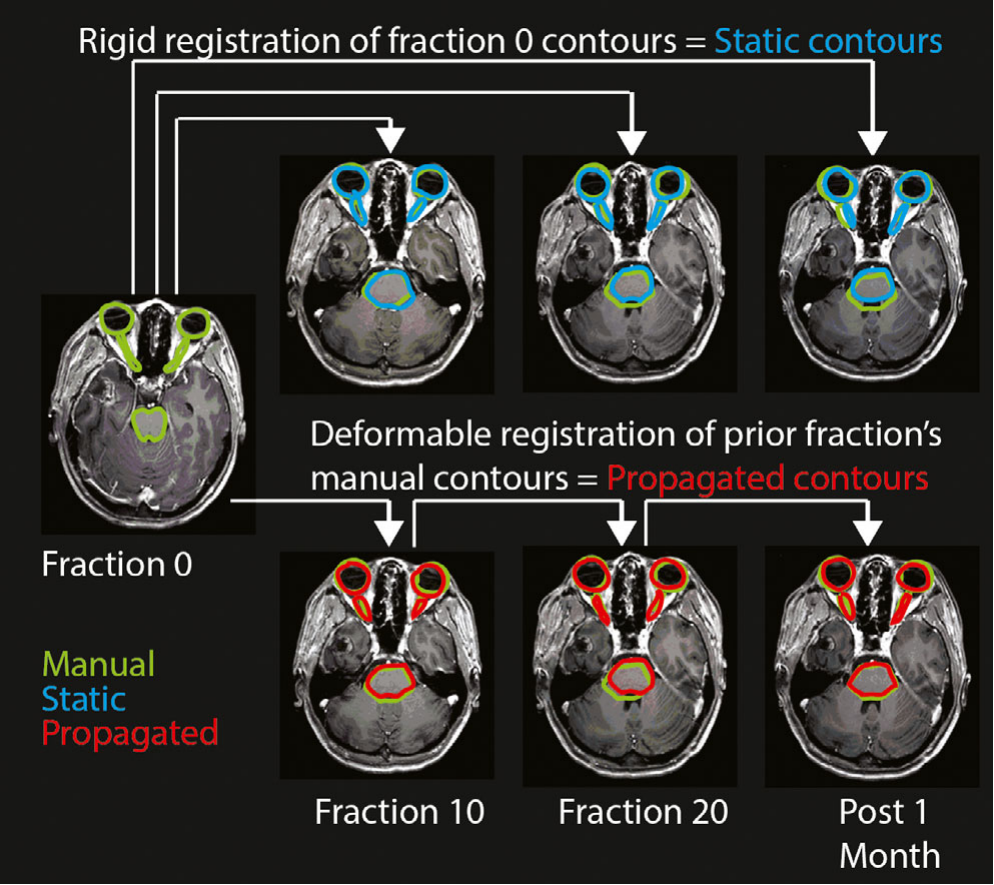
Static contours and propagated contours compared to manual contours. Manual contours (green) generated on each time point MRI were compared to static contours (blue) and to propagated contours (red). Static contours were the fraction 0 manual contours rigidly registered onto the MRI of the current fraction. Propagated contours were deformably registered from the manual contours of the prior time point. Arrows connect the baseline atlas to the resulting segmentation.

Manually contoured OARs from the planning scan were propagated to the MRIs of subsequent fractions using a deformable registration algorithm through ADMIRE software version v2.0.0.1, which was run from a research version of the Monaco treatment planning system version v5.19.03 (Elekta AB, Stockholm, Sweden). The current clinical version of Monaco in use on the MRI‐linac uses a deformable image registration algorithm that is identical to the atlas‐based algorithm used in the present study. The method simulated a semi‐automatic workflow in which the contours for the current time point are automatically segmented using the manual contours from prior time point as the reference atlas. Manual contours were propagated to the fraction 10 MRI with a single atlas segmentation method using the fraction 0 manual contours as the reference atlas. The segmentation involved several steps including linear registration, poly‐smooth nonlinear registration, and dense hybrid deformable registration as outlined in work by Han et al.^8^ The deformably registered contours were evaluated for accuracy by comparing to the manual contours. Similarly, the fraction 10 manual contours were used as a reference atlas to automatically segment the OARs on the fraction 20 MRI and the fraction 20 manual contours were used to automatically segment the OARs on the post 1 month MRI (Fig. 1). The proposed propagation scheme reflects an anticipated workflow with the MRL in which the contours are propagated each day and are manually corrected before the radiation plan is reoptimized and delivered.

### 2.3 Static contours vs manual contours

In order to assess the presence and degree of interfractional deformation in OARs during treatment, the manual contours of each fraction were compared to the manual contours of fraction 0. To make the comparison, the fraction 0 MRI was rigidly coregistered to the MRI of each subsequent time point and the resulting overlap between the manual contours and fraction 0 “static” contours was assessed using the Dice similarity coefficient (DSC) and Hausdorff distance (HD).^9^ For two three‐dimensional regions, A and B, the DSC measures the degree of overlap and is defined as

$$DSC=\frac{2\left|A\cap B\right|}{\left|A\right|+\left|B\right|},$$

where $\left|A\right|$ is the volume of region A, and $\left|A\cap B\right|$ is the volume of the intersection of regions A and B. If A and B perfectly overlap, DSC = 1. The Hausdorff distance measures the maximum distance from each point on A to the nearest point on B. More formally, denoting the distance between a point *a* on the contour of A and point *b* on the contour of B as $d\left(a,b\right)$, the HD is defined as

$$HD=\max_{a\in A}\left\{\min_{b\in B}\left\{d\left(a,b\right)\right\}\right\},$$

with perfectly overlapping contours giving HD = 0.

The impact of the changing OAR contours on dosimetry was determined by calculating the difference in the minimum dose to the 0.03 cc receiving the highest dose (D0.03 cc) on the manual contours vs the D0.03 cc on the static contours (referred to as ΔD0.03 cc).^10^ A positive ΔD0.03 cc denotes that the dose to the static contours is higher than the dose to the manual contours and vice versa for a negative ΔD0.03 cc. The clinically approved and delivered radiation plan (i.e., the reference plan which was not adapted during treatment) with a prescription of 6000 cGy in 30 fractions was used for dose determination on OARs at each time point on the Monaco treatment planning system. Dose to each OAR was calculated after the MRIs of each time point were rigidly registered to the planning CT which had a resolution of 0.88 × 0.88 × 1.0 mm. For simplicity, the cumulative dose to each of the OARs was calculated by applying the whole 6000 cGy in 30 fractions of the static radiation plan to the particular contour of interest.

### 2.4 Propagated contours vs manual contours

Similarly, the propagated contours of each time point were compared to the corresponding manual contours using DSC and HD metrics. The impact of contour accuracy on dosimetry was determined by calculating the difference in the minimum D0.03 cc on the propagated contours vs the D0.03 cc on the manual contours (ΔD0.03 cc). A positive ΔD0.03 cc denotes that the dose to the propagated contours is higher than the dose to the manual contours and vice versa for a negative ΔD0.03 cc. Dose to each propagated OAR was calculated after the MRIs of each time point were rigidly registered to the planning CT. The clinically approved and delivered static radiation plan was used for dose determination.

### 2.5 Comparison of static contours vs propagated contours

To compare the performance of the static contours vs the propagated contours, the manual contours were used as a ground truth. The DSC, HD, and ΔD0.03 cc between the static contours vs manual contours were compared to the DSC, HD, and ΔD0.03 cc between the propagated contours vs manual contours.

### 2.6 Statistical analysis

Statistical significance on the differences observed was determined using the Wilcoxon signed‐rank test, with significance level defined as *P* < 0.05. All statistical analyses were done on MATLAB and Statistics Toolbox (Release 2015, The MathWorks Inc., Natick, United States).

## 3 RESULTS

Serial MRIs were prospectively obtained on 14 recruited patients. Patients started treatment after a median of 25 (range 18–89) days following surgical resection as shown in (Table 1). Fraction 10, fraction 20, and post 1 month imaging was done after a median of 14 (range 13–19), 28 (range 25–33), and 74 (range 70–79) days following the planning scan (fraction 0).

**Table 1:** Patient characteristics.

Gender	
Male	5
Female	9
Age (yr)	
Median	50
Range	21–66
Time between STR and RT (d)	
Median	25
Range	18–89
Side	
Left	8
Right	6
Volume [median (range)] (cc)	
PTV	264 (136–341)
Lens	0.11 (0.02–0.21)
Eyes	9.15 (7.73–10.4)
Optic nerve	0.78 (0.60–1.02)
Optic chiasm	0.78 (0.57–1.17)
Brainstem	26.4 (22.2–33.2)

Abbreviations: PTV = planning target volume, RT = radiation therapy, STR = subtotal resection.

**Figure 2:**
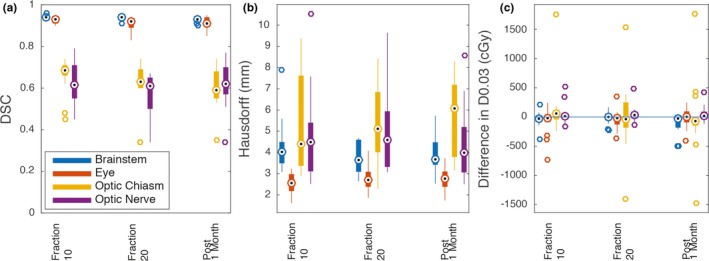
Fraction 0 contours vs manual contours. (a) DSC comparison between manual contours and static contours. There was a significantly different DSC for the optic chiasm between fraction 10 and fraction 20 (*P* = 0.04) and fraction 10 and post 1 month (*P* = 0.02). (b) HD comparison between manual contours and static contours. There were no significant differences in distance between each structure over time. (c) ΔD0.03 cc between manual contours and static fraction 0 contours. Brainstem dose was significantly different post 1 month (*P* = 0.004). Dotted circle denotes the median, edges of the bars are the 25th and 75th percentiles, the whiskers extend over mean ± 2.7 × standard deviation, and open circles denote outliers beyond the whiskers. Abbreviations: DSC = Dice similarity coefficient; HD = Hausdorff distance; ΔD0.03 cc = difference in the minimum dose to the 0.03 cc receiving the highest dose.

3.A Static contours vs manual contours:

The DSC between the static fraction 0 contours vs the manual contours from each fraction varied across all time points and structures with mean ± 1 SD of 0.77 ± 0.17. DSC for each structure across all patients and time points are summarized in Table 2. The HD between the static contours vs the manual contours had a mean ± 1SD of 3.96 ± 1.63 mm. The absolute ΔD0.03 cc between the static contours compared to the manual contours had a mean ± 1 SD of 137 ± 243 cGy. The mean DSC for the set of all structures significantly decreased over time (fraction 10: mean ± 1 SD 0.78 ± 0.17, post 1 month: mean ± 1 SD 0.76 ± 0.17, *P* = 0.02) while the mean HD did not increase significantly over time (*P* = 0.74) and the mean ΔD0.03 cc did not change significantly over time (*P* = 0.51). Changes for individual structures were not significantly different except for the DSC for the optic chiasm which significantly decreased over time (*P* = 0.04). The brainstem D0.03 cc was significantly lower post 1 month in the static contours compared to the manual contours (*P* = 0.004) (Fig. 2).

**Table 2:** Summary of performance comparisons between static fraction 0 contours vs propagated contours.

	Brainstem	Eye	Optic chiasm	Optic nerve
DSC				
Manual vs rigid	0.93 ± 0.001	0.92 ± 0.009	0.63 ± 0.004	0.60 ± 0.005
Manual vs propagated	0.94 ± 0.001	0.94 ± 0.003	0.68 ± 0.022	0.67 ± 0.008
*P*‐value	<0.001*	<0.001*	0.004*	<0.001*
HD (mm)				
Manual vs rigid	4.0 ± 0.26	2.7 ± 0.03	5.4 ± 0.27	4.8 ± 0.22
Manual vs propagated	3.7 ± 0.26	2.3 ± 0.07	5.9 ± 0.30	4.2 ± 0.56
*P*‐value	0.09	<0.001*	0.23	0.06
Δ D0.03 cc (cGy)				
Manual vs rigid	−56 ± 36	−45 ± 44	39 ± 100	46 ± 25
Manual vs propagated	−22 ± 28	−20 ± 54	−148 ± 14	−23 ± 45
*P*‐value	0.02*	0.8	<0.001*	<0.001*

Performance measures are shown as mean ± 1 SD. ΔD0.03 cc values shown are the difference of the static fraction 0 contours D0.03 cc minus the manual contours D0.03 cc or the propagated contours D0.03 cc minus manual contours D0.03 cc. Abbreviations: DSC = Dice similarity coefficient, HD = Hausdorff distance, D0.03 cc = minimum dose to the 0.03 cc receiving the highest dose.

* *P*‐value < 0.05.

### 3.1 Propagated contours vs manual contours

**Figure 3:**
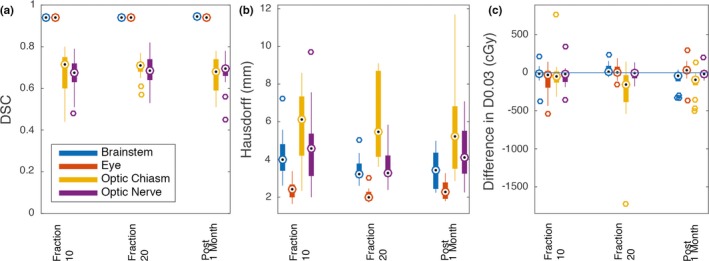
Propagated contours vs manual contours. (a) DSC comparison between manual contours and propagated contours. There was a significantly different DSC for the eye between fraction 10 and fraction 20 (*P* = 0.03). (b) HD between manual contours and propagated contours. There was a significantly different HD for the brainstem between fraction 10 and fraction 20 (*P* = 0.01). (c) ΔD0.03 cc between manual contours and propagated contours. Brainstem dose was significantly different post 1 month (*P* = 0.004). Dotted circle denotes the median, edges of the bars are the 25th and 75th percentiles, the whiskers extend over mean ± 2.7 × standard deviation, and open circles denote outliers beyond the whiskers. Abbreviations: DSC = Dice similarity coefficient; HD = Hausdorff distance; ΔD0.03 cc = difference in the minimum dose to the 0.03 cc receiving the highest dose.

The DSC between the propagated contours vs the manual contours varied across all time points and structures with mean ± 1 SD of 0.81 ± 0.15. The HD between the propagated contours vs the manual contours had a mean ± 1SD of 3.77 ± 1.81 mm. The ΔD0.03 cc between the propagated contours vs the manual contours had a mean ± 1 SD of 102 ± 159 cGy. The mean DSC for the set of all structures did not change significantly over time (*P* = 0.70). The DSC for the eye significantly increased from 0.938 to 0.944 from fractions 10 to 20 (*P* = 0.03). The mean HD for the set of all structures did not increase significantly over time (*P* = 0.40). The HD for the brainstem significantly dropped between fractions 10 to 20 (*P* = 0.013). The difference in D0.03 cc for the set of all structures did not significantly change over time (*P* = 0.57). The ΔD0.03 cc for the brainstem was significantly lower at post 1 month (*P* = 0.004) (Fig. 3).

### 3.2 Comparison of static contours vs propagated contours

**Figure 4:**
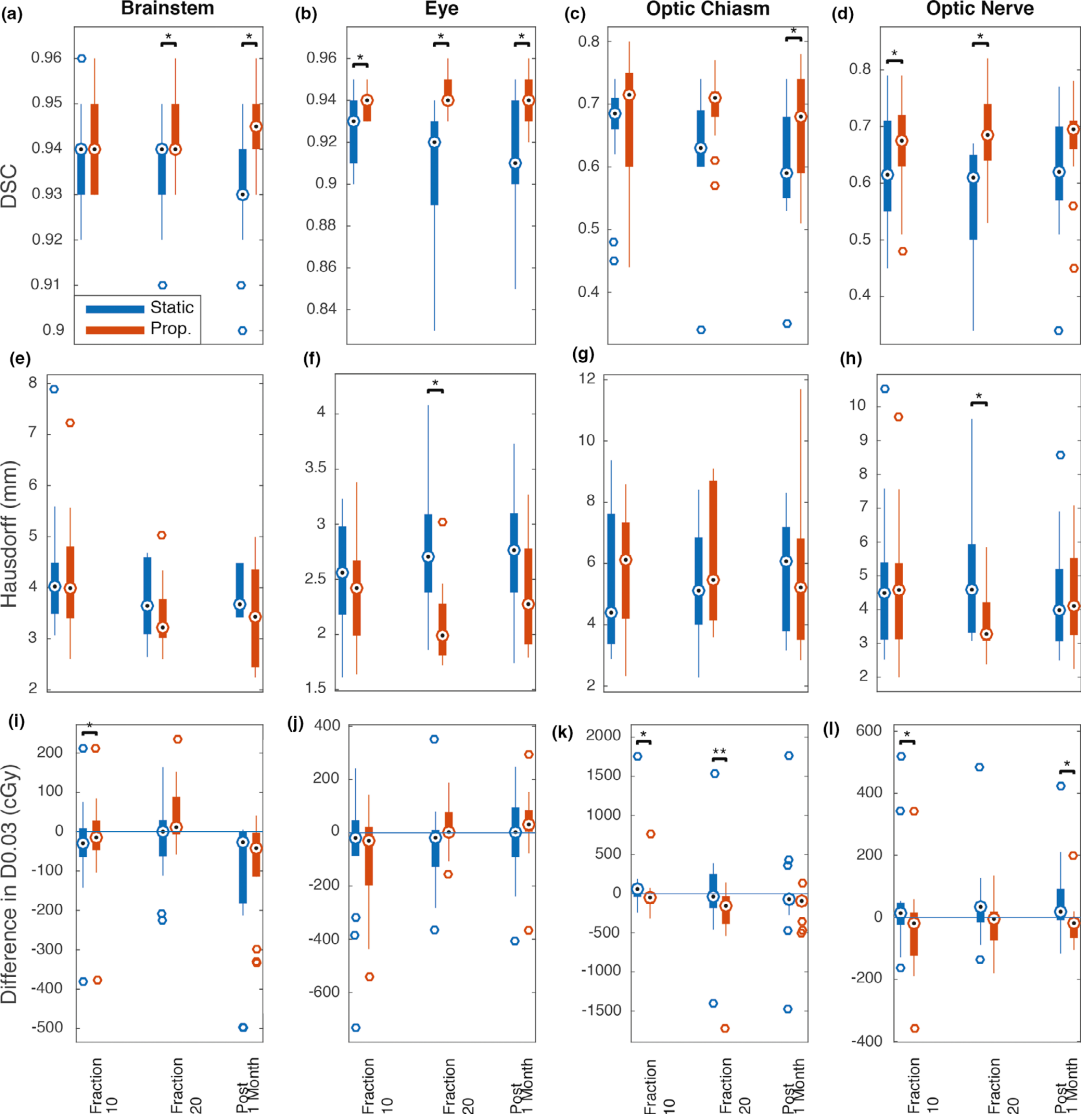
Comparison of static contours vs propagated contours. (a–d) DSC of the static vs propagated contours. (e–h) HD of the static vs propagated contours. (i–l) ΔD0.03 cc of the static vs propagated. Propagated contours performed significantly better than the static contours with higher DSC, lower HD, and smaller absolute ΔD0.03 cc at several time points (marked with *). Static contours had a significantly smaller ΔD0.03 cc for the optic chiasm at fraction 20 (marked with **). Dotted circle denotes the median, edges of the bars are the 25th and 75th percentiles, the whiskers extend over mean ± 2.7 × standard deviation, and open circles denote outliers beyond the whiskers. Abbreviations: DSC = Dice similarity coefficient; HD = Hausdorff distance; ΔD0.03 cc = difference in the minimum dose to the 0.03 cc receiving the highest dose.

Using the manual contours from each MRI as a ground truth, the propagated contours from the fractions 10 and 20 MRI and the post 1 month MRI were compared to the static contours from the fraction 0 MRI. For the set of all structures, the propagated contours had a significantly higher DSC compared to the static contours (propagated: mean ± 1 SD 0.81 ± 0.15, static: mean ± 1 SD 0.77 ± 0.17, *P* < 0.001). For individual structures, the DSC of the propagated contours was significantly (*P* < 0.05) higher than the DSC of the static contours for brainstem, eyes, optic chiasm, and optic nerve at 67% (8/12) of the time point comparisons. For the set of all structures, the propagated contours had a significantly lower HD compared to the static contours (propagated: mean ± 1 SD 3.77 ± 1.8 mm, static: mean ± 1 SD 3.96 ± 1.6 mm, *P* = 0.002). For individual structures, HD of the propagated contours was significantly (*P* < 0.05) lower than the HD of the static contours for the eyes and optic nerve at 17% (2/12) of the time point comparisons. For the set of all structures, propagated contours had a significantly lower absolute ΔD0.03 cc compared to the static contours (propagated: mean ± 1 SD 102 ± 159 cGy, static: mean ± 1 SD 137 ± 243 cGy, *P* = 0.019). For individual structures, the absolute ΔD0.03 cc of the propagated contours was significantly (*P* < 0.05) higher than the absolute ΔD0.03 cc of the static contours for the optic chiasm at 8.3% (1/12) of the time point comparisons and significantly (*P* < 0.05) lower for the brainstem, optic chiasm, and optic nerve at 33% (4/12) of the time point comparisons (Fig. 4).

## 4 DISCUSSION

In the present study, we found significant nonrigid changes in OARs in GBM patients on prospectively acquired serial MRIs during and after adjuvant CRT that lead to different maximum doses than planned. By using semi‐automatic OAR contour propagation, the OARs were more accurately delineated, which increased the accuracy of the reported dose to the OARs.

Using manual contours as a ground truth, the static contours had a significant decrease in DSC over time. In other words, the manual contours from fraction 0 were significantly different from the manual contours from subsequent fractions and 1 month post adjuvant CRT. While there were some significant changes in HD and difference in D0.03 cc for individual OARs, when all OARs were grouped and compared over time, only significant differences in DSC remained. The lack of significant changes in HD and D0.03 cc for the set of all OARs together was likely due to the small sample size. The change in OARs may be in part due to changes observed in the tumor, resection cavity volume, and associated intracranial edema during adjuvant CRT.^4^, ^5^, ^11^, ^12^ Compared to the static contours, the propagated contours had a significantly higher DSC, lower HD, and lower absolute ΔD0.03 cc, demonstrating that semi‐automatic contour propagation is able to track changes in the OARs. An exception to the above results favoring the propagated contours was the optic chiasm. This may be due to intra‐observer inconsistencies in the manual contours as it is often difficult to determine the anatomical borders of the structure in relation to the transition into optic nerves anteriorly and optic tracts posteriorly. In addition, for the set of all structures, the HD for both the propagated contours and the static contours was relatively large, in the range of around 4 mm. This may partly be due to the fact that the MRI slice thickness was 1.5 mm. A contour that is off by one or two slices, which is not uncommon for structures such as the brainstem, produces a HD of 1.5–3 mm. The uncertainty in the transition from the optic nerves to the optic tracts also likely contributed to the large HD.

Some of the results can be explained by the type of metric used. DSC is a reliable evaluation method for volumetric segmentations where high‐quality contours and a high degree of overall agreement is expected. HD is sensitive to outliers and is more reliable when the contours are small and overlaps are small.^9^ The results showed a greater number of significant differences in DSC between static and propagated contours compared to the number of significant differences using the HD metric as the OARs compared typically had a high degree of overlap. As shown in Fig. 4, for organs that had a high degree of overlap, such as the eye, the separation of the DSC between the static and propagated contours over time was clear. However, for organs that had a lower degree of overlap and greater uncertainty for the contour edges such as the optic nerve, the separation of the DSC between static and propagated contours over time was less obvious.

Delineation of OARs in the brain is detailed in guidelines such as those by Scoccianti et al and Niyazi et al.^13^, ^14^ However, despite such guidelines, inter‐observer variation in the delineation of OARs in the brain is significant.^15^, ^16^ The agreement between manual ground truth contours and both the static and propagated contours in this work was greater than the inter‐observer agreement in the literature. For example, the static contour mean DSC for the brainstem was 0.93 and for the optic nerve was 0.6 compared to the reported inter‐observer DSC of 0.83 and 0.5, respectively.^7^ Intra‐observer variability may also contribute to uncertainty of OAR delineation, with intra‐observer variability of 20% being quoted in brain tumor contouring.^17^

Both the intra‐ and inter‐observer OAR contour variation as well as the change in OARs over time results in a significant variation in the maximum dose estimates to the OARs. In previous studies, the inter‐observer variability contributed to a range of maximum doses to the optic apparatus equivalent to 70% of the prescribed dose in stereotactic radiosurgery.^15^, ^18^ In our study, the range of dose differences to the optic chiasm ranged from −1500 to 1700 cGy for a 6000 cGy plan, corresponding to a variability range of 53% of the prescribed dose. The wide variation in maximum dose in our study can be explained by the high‐dose gradients surrounding the OARs. Typical gradients in the high‐dose fall‐off region were 300–400 cGy/mm. A second contributing factor was the variability in the contouring of the chiasm, where it demonstrated the highest average HD compared to other OARs, with a mean of 5.9 mm for the propagated contours. Similarly, in a study for oropharyngeal carcinoma, inter‐observer contour variability contributed to a maximum dose increase of 23% to the brainstem.^16^ In our study, the greatest change in maximum dose to the brainstem was 400 cGy (7% of the maximum dose of 5400 cGy). Therefore, the variations in dose between the propagated contours and the ground truth observed in our study were smaller than the variations in dose due to inter‐observer variability studied in published literature.

Use of automatic tumor and OAR contouring can help reduce inter‐ and intra‐observer variability. Several algorithms can be used to automatically contour the OARs including atlas‐based methods, statistical models, and deformable models.^19^ The use of automatic tumor volume segmentation is an active area of research. Studies evaluating automated segmentation of the OARs and target volumes have shown that although automated segmentation can reduce the amount of time required to contour, manual editing of automated contours is still required.^20^, ^21^ In this study, we show the feasibility of semi‐automated contour propagation, using deformable registration of the manual contours from the prior time point. Using manually corrected semi‐automatically propagated contours is similar to our daily clinical workflow in which a radiation oncologist reviews and corrects the work of a resident, which has been reported previously.^22^

The ability to visualize and adapt radiation plans according to changing tumor and OAR volumes may result in greater therapeutic ratios. The majority of recurrences in GBM patients occur locally in the field treated with high‐dose radiation.^23^ The possibility of reducing local recurrences with dose escalation and concurrent temozolomide is being investigated in the phase 2 NRG BN001 trial.^24^ Inter‐fractional tracking of the OARs with MR‐guided adaptive radiation therapy could allow safer dose escalation or facilitate personalized isotoxic dose boosting strategies.^25^

To our knowledge, this is the first study documenting the change in OARs in patients undergoing adjuvant CRT for GBM. Our dataset consisted of manual contours of OARs on serial MRIs at four time points for 14 patients. This was also the first study to use contour propagation of OARs on serial brain MRIs.

Limitations of this study include several differences from the envisioned future clinical workflow of using an MR‐linac for daily or weekly radiation plan adaptation. This study included a limited number of inter‐fraction and posttreatment MRIs. Furthermore, only the T1‐weighted MRIs with Gadolinium‐contrast enhancement were examined. Future workflows with daily contrast administration would not be possible due to renal toxicity. A reference radiation plan at baseline was used for the dosimetric evaluation of each of the OARs. The dose to the various contoured versions of the OARs was determined by applying the full 6000 cGy plan to each OAR contour. Dose accumulation with deforming structures and plan adaptation according to daily OAR and target positioning are aspects that were not conducted in this study. Partial voluming and resampling errors between MRI and CT could contribute to contouring variability for smaller structures such as the chiasm or optic nerve and could be further quantified in future work. Given that there was only one set of ground truth manual contours of all the OARs for each patient at each time point, there were no inter‐observer comparisons. Finally, it is recognized that the sample size was limited to 14 patients and four time points per patient. However, it should be noted that this was a novel prospective imaging study in clinical patients with an aggressive tumor, who are usually symptomatic with a limited life expectancy. Thus, given the challenges of this patient population, the successful accrual of 14 patients who were able to complete all four imaging sessions could be considered a strength. Future studies will include larger patient datasets as prospective collection of new data continues. Other areas to be investigated include the comparison of different segmentation methods including using fraction 0 as an atlas for deformable registration to all subsequent fractions or combining the contours of completed fractions into a combined atlas for deformable registration to the next fraction. Clinically, the effect of dexamethasone on edema and potentially deformations in adjacent OARs would be of interest as another future area of investigation.

## 5 CONCLUSIONS

In conclusion, inter‐fractional MR imaging of GBM patients undergoing CRT shows nonrigid changes in the OARs. These changes are more accurately captured by a semi‐automatic atlas‐based deformable registration contour propagation method. The greater accuracy of the propagated contours results in more accurate maximum dose estimates to the OARs. Our work demonstrates the feasibility of semi‐automatic contour propagation for the use of MR‐guided adaptive radiation therapy with an MRL.

## CONFLICT OF INTERESTS

Dr. Arjun Sahgal has been an advisor/consultant with Abbvie, Merck, Roche, Varian (Medical Advisory Group), and Elekta (Gamma Knife Icon); ex officio Board Member to International Stereotactic Radiosurgery Society (ISRS); received honorarium for past educational seminars with Elekta AB, Accuray Inc, Varian (CNS Teaching Faculty), BrainLAB, and Medtronic Kyphon; research grant with Elekta AB; and travel accommodations/expenses by Elekta, Varian, and BrainLAB. Dr. Sahgal also belongs to the Elekta MR Linac Research Consortium, Elekta Spine, Oligometastases, and Linac Based SRS Consortia. Dr. Chia‐Lin Tseng has received travel accommodations/expenses by Elekta, honorarium for past educational seminars with Elekta, and belongs to the Elekta MR Linac Research Consortium. Dr. Ruschin is the co‐inventor of and owns associated intellectual property specific to the image guidance system on the Gamma Knife Icon. Dr. Sten Myrehaug has received research support: Novartis AG; Honorarium: Novartis AG, Ipsen. None related to this work.
